# A randomised, feasibility trial of a tele-health intervention for Acute Coronary Syndrome patients with depression ('MoodCare'): Study protocol

**DOI:** 10.1186/1471-2261-11-8

**Published:** 2011-02-25

**Authors:** Adrienne O'Neil, Anna L Hawkes, Bianca Chan, Kristy Sanderson, Andrew Forbes, Bruce Hollingsworth, John Atherton, David L Hare, Michael Jelinek, Kathy Eadie, C Barr Taylor, Brian Oldenburg

**Affiliations:** 1 School of Public Health and Preventive Medicine, Monash University, Melbourne, Australia; 2 Viertel Centre for Research in Cancer Control, Cancer Council Queensland, Brisbane, Australia; 3 School of Public Health, Queensland University of Technology, Brisbane, Australia; 4 Menzies Research Institute Tasmania, University of Tasmania, Hobart, Australia; 5 Centre for Health Economics, Monash University, Melbourne Australia; 6 The Royal Brisbane and Women's Hospital and Department of Medicine University of Queensland, Brisbane, Australia; 7 The University of Melbourne and The Austin Hospital, Melbourne Australia; 8 St. Vincent's Hospital, Melbourne, Australia; 9 Department of Psychiatry, Stanford University, Palo Alto, California, USA

## Abstract

**Background:**

Coronary heart disease (CHD) and depression are leading causes of disease burden globally and the two often co-exist. Depression is common after Myocardial Infarction (MI) and it has been estimated that 15-35% of patients experience depressive symptoms. Co-morbid depression can impair health related quality of life (HRQOL), decrease medication adherence and appropriate utilisation of health services, lead to increased morbidity and suicide risk, and is associated with poorer CHD risk factor profiles and reduced survival. We aim to determine the feasibility of conducting a randomised, multi-centre trial designed to compare a tele-health program (MoodCare) for depression and CHD secondary prevention, with Usual Care (UC).

**Methods:**

Over 1600 patients admitted after index admission for Acute Coronary Syndrome (ACS) are being screened for depression at six metropolitan hospitals in the Australian states of Victoria and Queensland. Consenting participants are then contacted at two weeks post-discharge for baseline assessment. One hundred eligible participants are to be randomised to an intervention or a usual medical care control group (50 per group). The intervention consists of up to 10 × 30-40 minute structured telephone sessions, delivered by registered psychologists, commencing within two weeks of baseline screening. The intervention focuses on depression management, lifestyle factors (physical activity, healthy eating, smoking cessation, alcohol intake), medication adherence and managing co-morbidities. Data collection occurs at baseline (Time 1), 6 months (post-intervention) (Time 2), 12 months (Time 3) and 24 months follow-up for longer term effects (Time 4). We are comparing depression (Cardiac Depression Scale [CDS]) and HRQOL (Short Form-12 [SF-12]) scores between treatment and UC groups, assessing the feasibility of the program through patient acceptability and exploring long term maintenance effects. A cost-effectiveness analysis of the costs and outcomes for patients in the intervention and control groups is being conducted from the perspective of health care costs to the government.

**Discussion:**

This manuscript presents the protocol for a randomised, multi-centre trial to evaluate the feasibility of a tele-based depression management and CHD secondary prevention program for ACS patients. The results of this trial will provide valuable new information about potential psychological and wellbeing benefits, cost-effectiveness and acceptability of an innovative tele-based depression management and secondary prevention program for CHD patients experiencing depression.

**Trial Registration Number:**

Australia and New Zealand Clinical Trials Register (ANZCTR): ACTRN12609000386235

## Background

Coronary Heart Disease (CHD) and depression are currently two of the most important causes of disability in high-income countries [1] and it is projected that the same will apply to low and middle income countries by 2030 [2]. These conditions often co-exist with approximately 15% of Myocardial Infarction (MI) patients experiencing major depressive disorder (MDD) and another 15-20% exhibiting mild to moderate depression [3]. Patients with post-MI depression are more likely to report impaired health related quality of life (HRQOL) [4], poorer medication adherence [5] and utilisation of health services [6], increased morbidity and suicide risk [7], and poorer CHD risk factor profiles, work outcomes [8] and survival [9]. In particular, depression results in poor uptake and completion of CHD secondary prevention or cardiac rehabilitation programs [10], which have been shown to play a pivotal role in improving CHD risk factor profiles and other clinical outcomes. Indeed, compared with non-depressed patients, depressed patients are three times less likely to be compliant with medical treatment recommendations [11]. The clinical benefits of CHD secondary prevention programs are well documented and include decreased risk of fatal and non-fatal recurrent MI and CVD [12], improved HRQOL, and lower rates of rehospitalisation [13,14]. However, despite the high prevalence of depression following a diagnosis of CHD and the poor outcomes associated with the group [9], it remains poorly recognised and managed in CHD patients.

Traditionally, CHD secondary prevention programs are delivered face-to-face in clinic- or hospital-based settings; however, they suffer from low participation rates due to a range of barriers including poor accessibility and access [15]. Symptoms of depression following Acute Coronary Syndrome (ACS) - including hopelessness, helplessness, and apathy - can further impede participation in secondary prevention programs. Contemporary approaches to CHD secondary prevention may help to overcome this treatment gap. A recent meta-analysis demonstrated that innovative, tele-based CHD secondary prevention programs may transcend some of the barriers to participation in traditional rehabilitation programs, and they are effective in improving behavioural and clinical outcomes for cardiac patients [16]. Further, telephone-delivered therapy has proven effective for patients with depression [17] and more recently, for those with co-morbid depression following a cardiac event. For example, a tele-health, nurse-delivered, collaborative care intervention for coronary artery bypass graft surgery patients suffering from depression significantly improved depression outcomes, mental health components of HRQOL and disease specific physical functioning [18].

Psychological based therapies [19,20], pharmacologic approaches (namely Selective Serotonin Reuptake Inhibitors)[21], and composite approaches to treatment [22,23] have all demonstrated improvements in depression for CHD patients, especially for those with recurrent depression [24]. While pharmacologic and psychological approaches have yielded comparable effect sizes in reducing depression [25], Cognitive Behaviour Therapy (CBT) has been shown to be particularly favourable for improving depression outcomes for cardiac patients, with an American Heart Association report endorsing its use [26]. Evidence from a number of well designed trials also demonstrate its effectiveness in reducing depression in cardiac patients when compared with other approaches [19]. For example, compared to usual care (UC), Freedland and colleagues (2007) demonstrated that CBT displayed greater and more durable effects than the other approaches [19]. However, relatively little is known about its effectiveness under 'real world' delivery conditions, particularly using a tele-based approach.

The feasibility of combining a tele-health, depression management program using CBT with a CHD secondary prevention program for ACS patients is yet to be established in the 'real world' setting. The effects of such a program could go beyond treating depression to improve all aspects of HRQOL and CHD risk factors, and demonstrate significant economic advantages over more traditional modes of delivery. This paper presents the study protocol for a randomised, multi-centre, feasibility trial of a tele-health intervention for ACS patients with depression ('MoodCare'). We hypothesise that the trial will demonstrate the feasibility of the MoodCare intervention through improving key depression and HRQOL outcomes at 6 months, with increased participant satisfaction, and will be cost-effective compared with UC.

## Methods

We are currently enrolling 100 ACS patients in a prospective, multi-centre, feasibility trial with patients randomised to either the intervention or UC control group. Participants in both groups complete assessments at baseline (Time 1), post-intervention or 6 months follow-up (Time 2), as well as at 12 months (Time 3), and 24 months (Time 4) for longer term effects.

### Study Aims

#### Primary Aim

To investigate the feasibility of a telephone-delivered, depression management and CHD secondary prevention intervention for ACS patients over 6 months. Prospective outcomes include: changes in depression using the Cardiac Depression Scale (CDS) and changes in HRQOL using the Short Form-12 (SF-12), cost-effectiveness and participant acceptability compared with UC.

#### Secondary Aims

(i) To investigate the longer term feasibility of implementing the MoodCare program at 12 and 24 months follow-up.

### Study Sample

#### Eligibility Criteria

Eligibility criteria includes: a clinical diagnosis consistent with that of ACS (MI [STEMI or non STEMI] or unstable angina confirmed by angiogram), aged 21-85 years, fluency in English, availability via the telephone for the duration of the study, and a Patient Health Questionnaire (PHQ9) score of 5-19. Patients are excluded if they are: participating in regular psychological therapy with a mental health professional at the time of admission for ACS, diagnosed with a psychiatric condition which may impact on involvement (including bipolar disorder, psychotic illness of any type, dementia, acute suicidality, severe personality disorder), cognitively impaired impacting on their ability to participate in the study, diagnosed with a terminal illness, or unable to participate in a tele-based unsupervised mood and lifestyle intervention as confirmed by the treating clinician.

#### Sample Recruitment Procedures

We are screening more than 1600 patients over approximately 15 months from six metropolitan hospitals in Victoria and Queensland, Australia. Recruitment commenced in December 2009. All consenting patients are assessed for depression prior to hospital discharge using a psychometrically robust and valid instrument (PHQ9) [27]. Patients with a PHQ9 score of 5-19 are eligible to participate. This scoring range was selected due to its high sensitivity and specificity, as opposed to the commonly used cut off of ≥ 10, which has comparable specificity (92% and 90%, respectively), but poorer sensitivity (39% and 54%, respectively) [28]. Patients with a PHQ9 score <5 are provided with relevant feedback, reassurance and advice. Any persons indicating suicidal thoughts on PHQ9 and/or those with severe depression, as indicated by PHQ9 scores of 20-27, are referred for further assessment by a mental health professional and possible study exclusion. Algorithms are in place for patients assigned to the intervention whose condition deteriorates throughout the program, and who are no longer eligible or lost to follow up throughout the course of the trial.

Eligible participants are subsequently contacted by the research team via telephone within 1-2 weeks of discharge to complete Time 1 data collection. This includes a secondary assessment of depression in order to determine severity. Participants initially respond to a two item screening tool which has a positive endorsement of the first two PHQ items [28], before undertaking the full Composite International Diagnostic Interview (CIDI) assessment.

#### Sample Size Calculations

Anticipating an attrition rate of 20% throughout the study, n = 125 participants are required to achieve a final sample size of n = 100. Sample size analysis indicated that 50 subjects per group (intervention and control) or a total of 100 are required to complete the study in order to detect an absolute intervention effect with 80% power and type I error of 5% (two-tailed). Sample size was calculated based on an overall difference between participants in the intervention and control groups in the primary outcome measure of depression scores at 6 months, where a sample size of n = 100 was sufficient to detect a difference in mean CDS scores of 6.8, assuming a between-patient SD of 12 [29]. Assuming persistence of effect to 12 months and a conservative correlation of 0.30 between baseline, 6 months and 12 months CDS scores, a pooled analysis at 6 and 12 months will have 80% power to detect a difference of 5.1 units (a change score of 5 on the CDS is considered clinically meaningful).

### Ethics Approval

Ethics approval was received from Human Research Ethics Committees of St Vincent's Hospital, The Austin Hospital, The Royal Melbourne Hospital, The Geelong Hospital, The Prince Charles Hospital, The Royal Brisbane and Women's Hospital, and Monash University.

### Study conditions

Both control and intervention participants receive a brief National Heart Foundation of Australia education pamphlet on MI recovery and a biennial study newsletter based on existing educational materials to enhance study retention. On enrolment, a letter is sent to all participants' primary care provider/s informing them of the aims of the study, the group to which the patient has been randomised and other relevant information that may be required from the participant and the care provider at follow-up.

#### Control

Control participants continue to receive their usual medical care through their health care providers.

#### Intervention

The intervention commences within two weeks of baseline screening and is delivered by qualified psychologists ('counsellors') from Monash University. The intervention aims to manage depression as well as CHD risk factor behaviours using a tele-based care management model [30] incorporating CBT counselling [31]. Two psychologists with at least 2 years of relevant clinical experience with CBT are delivering the intervention. The counsellors provide information to patients via the telephone during structured counselling sessions and assist participants to set and attain specific short and long term goals and a plan of action to improve their mental health and CHD risk factor profiles. Cognitive restructuring, behavioural activation, goal setting and motivational interviewing techniques are utilised. Participants are encouraged to seek appropriate treatment and attend follow-up with their usual health care providers as necessary, in addition to activating social/family support and community and environmental linkages (e.g. employment, housing support). The intervention comprises up to 10 × 30-40 minute sessions over a six month period as CBT has been found to effectively reduce depressive symptoms over this time frame [22]. The sessions are most intensive over the first three months when depressive symptoms are most likely to affect patients following ACS [32] and to impact on their HRQOL and adoption of positive CHD risk factor behaviours. More frequent sessions may be scheduled during the treatment phase based on a patient's needs and interest but the intervention is limited to up to 10 sessions in total.

Intervention participants receive a handbook containing both project specific and general health resources. For example, the handbook contains monitoring forms and recording sheets to be used during and in between sessions for tracking the participant's mood, session activities, the participant's thoughts, CHD risk factor goals and changes. It provides additional information and details on the nature and treatment of depression, as well as the CBT model. More generally, supplementary materials including a list of health related resources are provided in the handbook.

### Study Integrity

The study design is guided by the CONSORT statement [33] and randomisation to study group occurs following the completion of Time 1 data collection. Project staff who are collecting data are blinded to participants' group allocation. Participants are asked not to reveal the group to which they have been randomised when completing data assessments. Stratified randomisation occurs using a separate block randomisation list that has been generated for each study group or strata. Randomisation is integrated into the web-based database and automatically generated following the conclusion of baseline assessment. The process is concealed from investigators. The schedule is stratified by severity of depression assessed by the CIDI (i.e. those meeting current diagnosis for depression versus those who do not). The intervention protocol is detailed in a manual. In addition to internal peer review, all intervention calls are audio-taped with a proportion reviewed by a senior clinical psychiatric consultant, using a standardised inventory in order to ensure clinicians' adherence to the delivery of the intervention protocol and treatment integrity. To assess the quality of the delivery of the intervention, a separate inventory based on the Cognitive Therapy Scale [34] has been developed for the MoodCare intervention. All data analyses are conducted on the basis of intention to treat.

### Data collection and outcome measures

Clinical and anthropometric data are collected at the time of the participant's initial screening, from hospital medical records (blood pressure, cholesterol level, haemoglobin A1C (HbA1c), family history of heart disease, height, weight, waist circumference, body mass index (BMI), procedures performed or scheduled, General Practitioner (GP) details, cardiologist details, whether patient was transferred from another hospital, details of initial admitting hospital, admission and expected discharge date). At follow-up, participants are requested to collect medical information (blood pressure, cholesterol level, HbA1c, weight, waist circumference) from their primary care provider/s medical records prior to Computer Assisted Telephone Interview (CATI). Self-report and CIDI interview data are collected, at Time 1, Time 2, Time 3 and Time 4 by CATI. Additional outcome data are collected via postal survey to minimise participant burden (see Table 1). The feasibility of the trial will be determined by the following outcome measures:

**Table 1:** Measurement of outcome variables at baseline (Time 1), post-intervention (Time 2), 12 months (Time 3), 24 months follow-up (Time 4)

Variable	Measurement tool	Inpatient Screening	1-2 weeks post discharge TIME 1	6 Months TIME 2	12 Months TIME 3	24 months TIME 4	Type of administration
**Depression**	Patient Health Questionnaire 9	✓	✓	✓	✓	✓	CATI^+^

	Two item screener [28]		✓				CATI

	Cardiac Depression Scale		✓	✓	✓	✓	CATI

	Composite International Diagnostic Interview		✓		✓	✓	CATI

**HRQOL**	Short Form 12		✓	✓	✓	✓	CATI

**Physical activity**	Active Australia Survey		✓	✓	✓	✓	Postal survey*

**Saturated Fat Intake**	Short Fat questionnaire		✓	✓	✓	✓	Postal survey*

**Social Support**	ENRICHD Social Support Inventory		✓	✓	✓	✓	CATI

**Smoking**	Cancer Council Food Frequency Questionnaire		✓	✓	✓	✓	Postal survey*

**Alcohol**	Cancer Council Food Frequency Questionnaire		✓	✓	✓	✓	Postal survey*

**Medication**	Self report/General Practitioner/data linkage		✓	✓	✓	✓	Postal survey,* medical records

**Anxiety**	General Anxiety Disorder-7		✓	✓	✓	✓	CATI

**Medical co-morbidities**			✓	✓	✓	✓	CATI

**Biomedical and anthropometric measurements**	Weight, height, waist circumference, Blood Pressure, lipids, fasting capillary blood glucose	✓			✓	✓	Medical records

**Employment**	Employment status, absenteeism/productivity		✓	✓	✓	✓	CATI

CATI = Computer Assisted Telephone Interview; *An envelope containing self administered paper copies of each instrument is posted to the participant prior to their baseline telephone interview. The survey is to be completed on approximately the same day as baseline CATI interview to ensure consistency of information, and returned to the Project Manager. ^+ ^In patient screening conducted face to face by nurses, using the PHQ9. Follow up PHQ9 administered via CATI at all assessment points thereafter

#### Changes in key depression and HRQOL outcomes

The two primary outcomes are changes in depression and HRQOL which are assessed using the CDS [35] and SF-12 [36], respectively. The CDS was originally designed to measure depressed mood over the full range generally seen in cardiac patients, combining excellent test-retest reproducibility with responsiveness to change over time [29]. In addition, it also has excellent sensitivity (97%) and appropriate specificity (85%) for the categorical diagnosis of major depression [37]. Other outcome variables include: physical activity [38], saturated fat intake [39], smoking, alcohol intake [40], anxiety [41], social support, employment, lipid profile, blood pressure, BMI, waist circumference, diabetes management (HbA1c), and pharmacological management (self report, health care utilisation data). Six month follow up will be the primary assessment point. The measures for each of these outcome variables are summarised in Table 1.

#### Cost-Effectiveness Analyses

A cost-effectiveness analysis (CEA) of the costs and outcomes for patients in the intervention and control groups will be undertaken. Detailed data on costs of Health Care Utilisation (HCU), access to community and other resources and medication are being collected from both the intervention and 'usual care' control groups. All resources utilised are costed using nationally applicable cost data (e.g. Diagnostic Related Groups costs for hospital admissions). Total medication costs are taken from patient contributions and estimated total Pharmaceutical Benefits Scheme benefits paid by the Health Insurance Commission. For HCU, data linkage with Medicare Australia's database allows the collection of health care utilisation data for use of items under Medicare and Pharmaceutical Benefits Scheme (PBS). Participants are requested to record current medications and number of general practitioner visits prior to Time 3 and Time 4 follow-up assessments.

The expected cost of readmission for ACS is also calculated using hospital-based costs and patient-specific probabilities of readmission for ACS computed using APACHE III (Acute Physiology And Chronic Health Evaluation III). The primary health outcome for the CEA is quality-adjusted life years (QALYs). These are calculated for both arms using HRQOL scores collected in the trial using the SF-6D, a derivative of the SF-12. The SF-12 instrument is widely used internationally, and has been recommended as appropriate and sensitive to change for CHD patients and in depression treatment [42]. For all economic analyses, detailed probabilistic sensitivity analysis will be undertaken for all projections and parameters with uncertainty and/or variability.

#### Intervention Implementation and Acceptability

To assess program implementation, participant acceptability is measured by a self-administered questionnaire at Time 2. Self-administered satisfaction questions include: 'how would you rate the MoodCare Participant Handbook' (*excellent *to *poor*); 'how would you rate your sessions with the Counsellor (*very useful *to *not useful at all)*; 'did you get what you expected from the program' (*yes definitely *to *no definitely not)*; 'to what extent has the program met your needs' (*almost all of my needs have been met *to *none of my needs have been met); *'has the program helped you to deal more effectively with your health issues' (*yes it helped a great deal *to *no it seemed to make things worse)*; 'in general how satisfied are you with the program (*very satisfied *to *very dissatisfied)*; 'were you satisfied with the length of the counselling sessions' (*yes, no)*; 'were you satisfied with the number of counselling sessions' (*yes, no)*; and 'were you satisfied with the length of the program overall' (*yes, no)*. Participants are also asked to highlight the strengths and weaknesses of the program in two open-ended questions. Participant adherence to the intervention is assessed by: the proportion of sessions completed during the intervention period; the topics covered in each session; and the total length (minutes) of intervention exposure during the six month period.

#### Long term maintenance and sustainability

The RE-AIM framework has been developed to evaluate the strengths and weaknesses of different approaches to chronic disease management [43]. This framework will be applied to further assess the feasibility of the MoodCare program. For example, we will evaluate the *Reach*, or representativeness of the sample by assessing the characteristics of eligible, enrolled and randomised participants. Similarly, to assess *Adoption*, characteristics on program uptake and adherence will be provided. The long term *Maintenance *of the program will be assessed by evaluating participant outcomes beyond six month follow up (e.g. 12, 24 months).

### Data Analyses

Baseline characteristics will be compared across intervention arms using summary statistics. Principal analyses will involve comparison of CDS and HRQOL outcomes at each time point between intervention arms using the baseline of the outcome as a covariate where relevant. Pooled analyses across all time points will employ linear mixed models. This will assess comparative rates of change over time, variability between rates of individuals, and allows for incomplete data due to a process of missingness where systematic dependence is upon observed covariates or outcomes, the so called "missing at random" assumption. Results will be expressed as estimated mean changes in CDS depression measures and HRQOL, as an overall mean excess intervention over usual care effect, all with corresponding 95% confidence intervals.

## Discussion

Depression often remains unrecognised and untreated in cardiac patients, despite increasing the risk of poor patient outcomes such as reduced HRQOL and increased mortality (including suicide). For the first time, this study will evaluate the feasibility, efficacy and cost-effectiveness of a state-of-the-art, tele-based depression management and CHD secondary prevention program for depressed ACS patients. If feasible and successful in promoting depression and HRQOL benefits, the intervention could provide an alternative for those who cannot tolerate or would prefer a non-pharmacological alternative to depression treatment. Further, this contemporary tele-health approach to management may help overcome some of the barriers to participation that are associated with face to face rehabilitation programs, including distance, cost and time. The intervention could be translated into clinical practice through community-based telephone helplines widely used in Australia and internationally or through acute clinical care settings.

## List of abbreviations

CHD: Coronary Heart Disease; MI: Myocardial Infarction; MDD: Major Depressive Disorder; HRQOL: Health Related Quality of Life; ACS: Acute Coronary Syndrome; CBT: Cognitive Behaviour Therapy; UC: Usual Care; CDS: Cardiac Depression Scale; SF-12: Short Form-12; STEMI: ST elevation Myocardial Infarction; Non STEMI: Non-ST elevation Myocardial Infarction; PHQ9: Patient Health Questionnaire; CIDI: Composite International Diagnostic Interview; CONSORT: Consolidated Standards of Reporting Trials; HbA1c: Haemoglobin A1C; BMI: Body Mass Index; GP: General Practitioner; CATI: Computer Assisted Telephone Interview; CEA: Cost-effectiveness analysis; HCU: Health Care Utilisation; PBS: Pharmaceutical Benefits Scheme; APACHE III: Acute Physiology And Chronic Health Evaluation III; QALYs: Quality-adjusted life years

## Competing interests

The authors declare that they have no competing interests.

## Authors' contributions

ALH, CBT, KS, DLH, BH, JA, MJ and BO developed the study concept and aims. BC, AO, BO, KE and ALH co-wrote the study protocol. BC, AO, CBT, KS, ALH and BO are implementing the study protocol and overseeing the collection of data. AF performed the sample size calculations and randomisation schedule and will oversee data analysis. AO and ALH drafted the study manuscript and all authors contributed to, read and approved the final manuscript.

## Authors' information

Dr Adrienne O'Neil can be contacted at the following email: adrienne.o'neil@med.monash.edu.au.

## Pre-publication history

The pre-publication history for this paper can be accessed here:

http://www.biomedcentral.com/1471-2261/11/8/prepub
